# Intraoral localized methotrexate‐associated lymphoproliferative disorders concurrent with antiresorptive agent‐related osteonecrosis of the jaw: A case report and literature review

**DOI:** 10.1002/ccr3.3192

**Published:** 2020-09-13

**Authors:** Masaki Minabe, Taiki Suzuki, Masumi Komatsu, Kazuhiko Hashimoto, Takeshi Nomura

**Affiliations:** ^1^ Department of Oral Medicine, Oral and Maxillofacial Surgery Tokyo Dental College Ichikawa Japan; ^2^ Division of Oral and Maxillofacial Surgery SUBARU Health Insurance Society Ota Memorial Hospital Ota Japan; ^3^ Department of Pathology and Laboratory Medicine Tokyo Dental College Ichikawa General Hospital Ichikawa Japan

**Keywords:** antiresorptive agent‐related osteonecrosis of the jaw, denosumab, methotrexate‐associated lymphoproliferative disorders, rheumatism, rheumatoid arthritis

## Abstract

Intraoral localized methotrexate‐associated lymphoproliferative disorders can cause antiresorptive agent‐related osteonecrosis of the jaw associated with infection due to its immunological abnormalities and ulcer formation.

## Graphical abstract

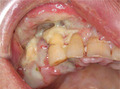

## 1 INTRODUCTION

Intraoral localized methotrexate‐associated lymphoproliferative disorders (MTX‐LPD) may be associated with antiresorptive agent‐related osteonecrosis of the jaw (ARONJ), especially with purulent discharge. In patients who are on oral MTX and have oral ulcers, it is important to perform biopsy, even if they meet the diagnostic criteria for ARONJ.

Methotrexate (MTX) is the first‐line treatment for rheumatoid arthritis (RA), as it has superior bone destruction suppressant effect.^1^ However, there are many reports on the adverse effects of MTX therapy. Several recent case studies reported that long‐term MTX therapy resulted in MTX‐related lymphoproliferative disorder (MTX‐LPD) in RA patients.^2^, ^3^

The World Health Organization categorizes MTX‐LPD as an “other iatrogenic immunodeficiency‐associated LPD” in the classification of Tumors of Hematopoietic and Lymphoid Tissues.^4^ Immunosuppression by MTX is thought to reduce the host immunosurveillance of the Epstein‐Barr virus (EBV)‐infected B cells, as approximately 50% of MTX‐LPD patients were EBV positive,^5^, ^6^ and lead to MTX‐LPD.

Antiresorptive agents, such as bisphosphonates (BPs) and denosumab, are the primary treatment agents for osteoporosis, bone metastasis, and hypercalcemia. Antiresorptive agents have many benefits for patients with skeletal complications. The use of corticosteroid and MTX is important for immunosuppression in RA treatment, and antiresorptive agents are important to prevent steroidal osteoporosis. In proportion to their use, cases of antiresorptive agent‐related osteonecrosis of the jaw (ARONJ) have been increasing.^7^ The major clinical symptoms of ARONJ are progressive destruction of the jaw, prolonged pain, bone exposure, soft tissue swelling, fistula, and infections. Most of those symptoms are intractable, and many severe cases have been reported.^8^, ^9^, ^10^ Therefore, the diagnosis and treatment of ARONJ should be made early.

Here, we report a case of MTX‐LPD in an elderly woman who had concurrent exposure of the maxilla, mucosal necrosis, and persistent pain from ARONJ caused by long‐term denosumab to treat steroidal osteoporosis of RA. In addition, we performed a literature review to identify all cases of intraoral localized MTX‐LPD with bone exposure and to investigate the characteristics and prognoses of these cases.

## 2 CASE REPORT

Patient: A 75‐year‐old woman.

Chief complaint: Persistent pain in the right maxillary mucosa and bone exposure.

Medical history: The patient had RA, osteoporosis, renal cancer, and schizophrenia. She had been taking 8 mg of MTX per week since 1998. Further, she was given denosumab for osteoporosis from 2013 to January 2018 (60 mg subcutaneously once every 6 months; the total dose administered was 600 mg). She underwent left nephrectomy for renal cancer in June 2017. She was undergoing medical therapy for schizophrenia.

Family history: No relevant family history.

Clinical procedures and outcomes: At her first visit to a dentist with pain in the right maxillary mucosa in early February 2018, the dentist suspected ARONJ because of maxillary posterior bone exposure, infection, and pain. She visited our department for further examination and treatment of the right maxillary lesion that was causing her pain in mid‐February 2018. Her dental treatment and tooth extraction history were unknown.

### 2.1 Presenting symptoms

General condition: The physical constitution was slightly obese. Oral intake of food was impaired because of severe intraoral pain.

Facial condition: The countenance was symmetric. The cervical lymph nodes were not swollen.

**FIGURE 1:**
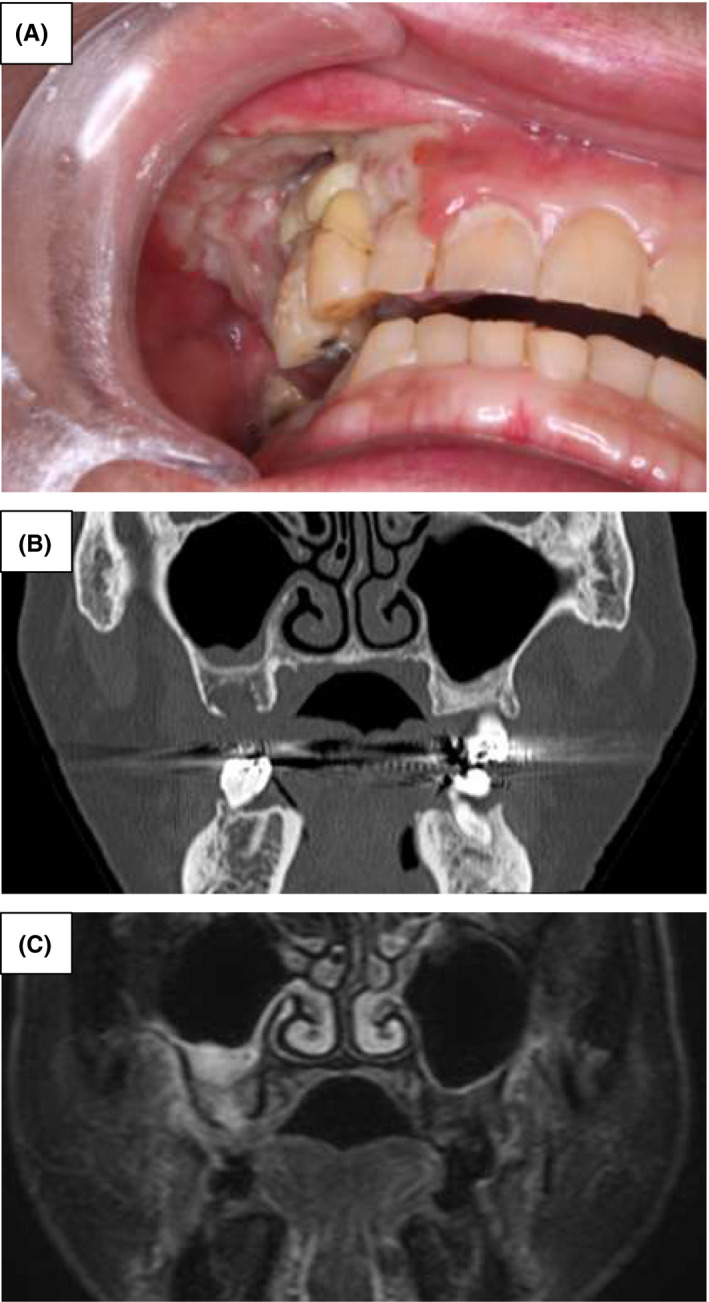
Oral cavity at the initial examination. A, Large ulceration with no peripheral induration, infection with purulent discharge, and exposed bone in the ulcer were noted on the right maxillary gingiva. B and C, Computed tomography and magnetic resonance imaging showed osteosclerosis in the right maxillary jaw bone, and the upper right maxillary sinus mucosa was hypertrophic

Oral condition: There were jaw bone exposure, mucosal necrosis, and infection from the right maxillary incisors to the molars. The lesion gave her severe pain (Figure 1A). The right maxillary canine and lateral incisor were loose, suggesting moderate to severe periodontitis.

Image findings: Computed tomography (CT) and magnetic resonance imaging (MRI) revealed osteosclerosis in the right maxillary jaw bone, and the upper right maxillary sinus mucosa was hypertrophic, suggesting acute osteomyelitis (Figure 1B and C).

Blood test findings: White blood cell count, 5100/μL; C‐reactive protein, 1.35 mg/dL; creatinine, 1.04 mg/dL; estimated glomerular filtration rate, 20%. It suggested slight inflammation and renal function impairment.

We performed cytodiagnosis and biopsy at the right maxillary mucosa on the first medical examination day. She was diagnosed negative for intraepithelial lesion and malignancy on cytodiagnosis with granulation tissue on histopathology.

Clinical diagnosis: ARONJ.

We initiated treatment for ARONJ, based on the treatment strategies in previous papers,^11^ with oral amoxicillin (750 mg/d), oral clarithromycin (400 mg/d), and the use of gargle for infection control.

**FIGURE 2:**
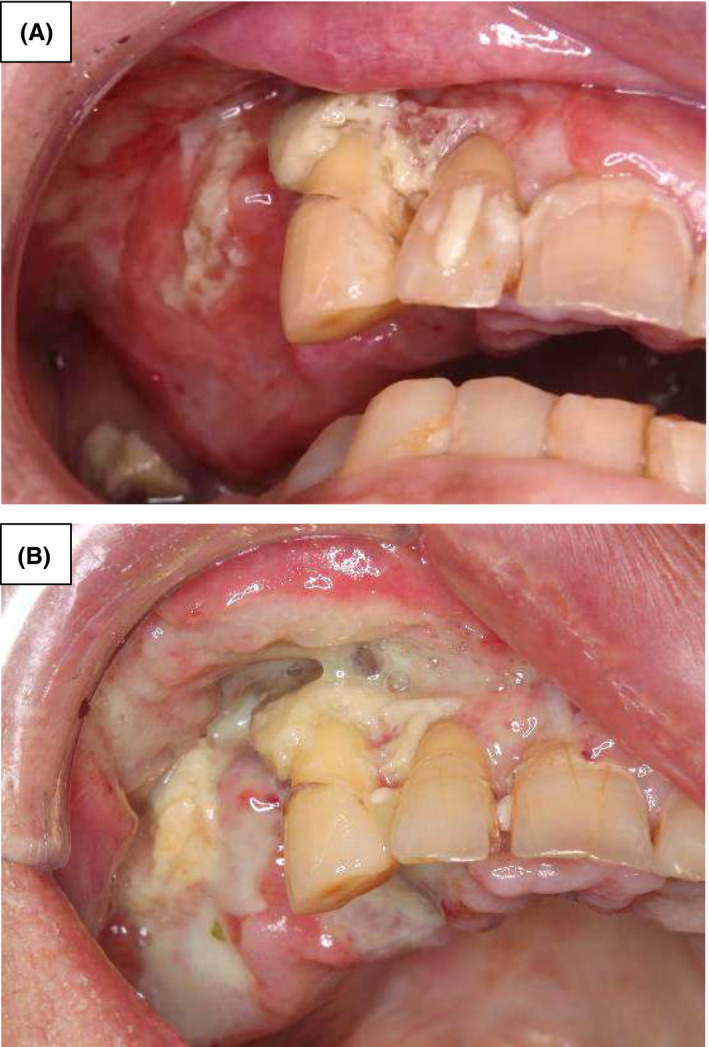
Oral findings after the initial treatment. A, Inflammatory findings had undergone remission on hospital day 18 after antimicrobial administration. B, The maxillary jaw bone exposure and mucosal necrosis had re‐spread on hospital day 65

**FIGURE 3:**
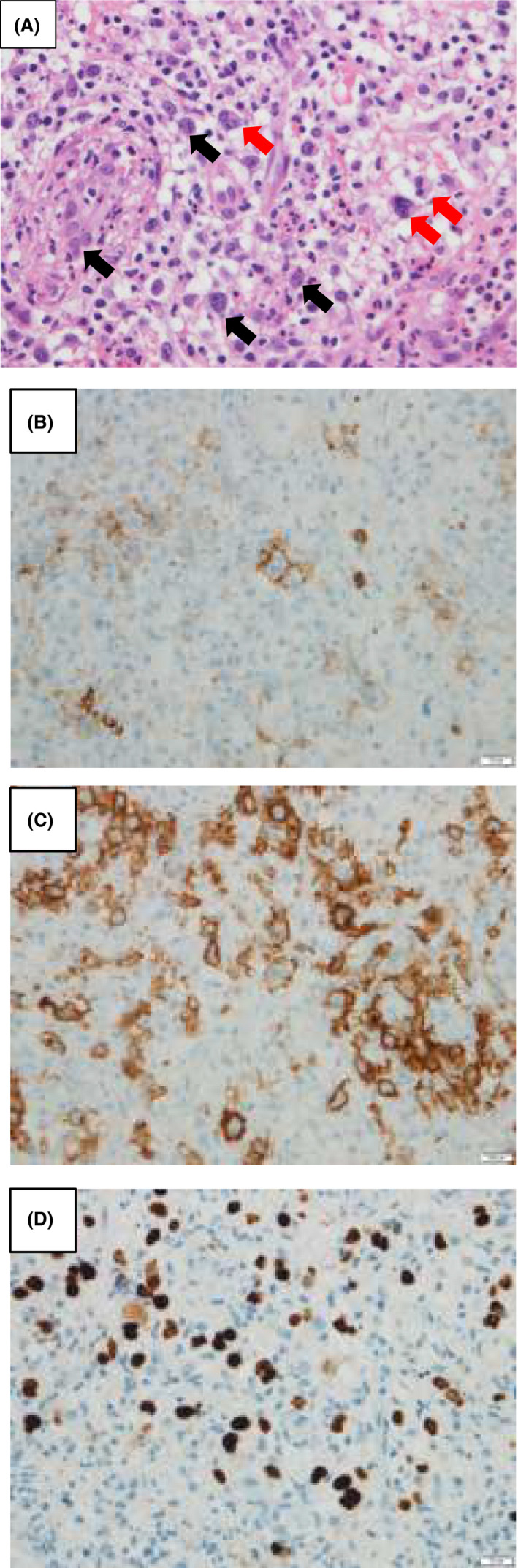
Histopathological and immunohistochemical findings. A, Histopathological staining with hematoxylin and eosin (×400) revealed severe inflammatory cell infiltration in the submucosal area. Granulation tissue in the deep subepithelial region, Reed‐Sternberg‐like cells (Red arrow), and Hodgkin cell‐like cells (Black arrow) were found in the granulation tissue. B‐D, Immune‐histological findings revealed many large CD20 (×400) (B) and CD30 (×400) (C)‐positive cells, which were positive for EBER‐ISH (×400) (D)

On hospital day 18, the inflammatory findings had undergone remission (Figure 2A). However, on hospital day 65, the maxillary jaw bone exposure and mucosal necrosis were found to have spread (Figure 2B). Therefore, we suspected MTX‐LPD because of the long‐term administration history and rise in blood concentration of MTX because of renal function impairment after nephrectomy. Therefore, we performed re‐biopsy at the right maxillary mucosa. On hospital day 72, we diagnosed MTX‐LPD using several immune‐histological examinations. Histopathological examination with hematoxylin and eosin staining revealed severe inflammatory cell infiltration in the submucosal area. Granulation tissue in the deep subepithelial region showed Reed‐Sternberg‐like cells and Hodgkin cell‐like cells. Immunohistochemical staining revealed several large CD20‐ and CD30‐positive cells, which were positive for EBER‐ISH (Figure 3).

We consulted the Department of Hematology and Rheumatology in our hospital to look for more lesions and also to replace MTX with other agents for RA. The malignant lymphoma tumor marker test showed that lactate dehydrogenase (277 IU/L) and soluble interleukin‐2 receptor (862 U/mL) were slightly elevated, but there were no findings of lymphoid nodal lesions on contrast‐enhanced CT. Finally, she was diagnosed with intraoral localized MTX‐LPD with ARONJ.

Final diagnosis: MTX‐LPD (intraoral localized type) with ARONJ stage 2, which is characterized by exposure/necrosis associated with pain, infection, and fistula, in which bone is palpable with a probe.

On hospital day 72, in consultation with the Department of Hematology and Rheumatology, MTX was switched to salazosulfapyridine.

**FIGURE 4:**
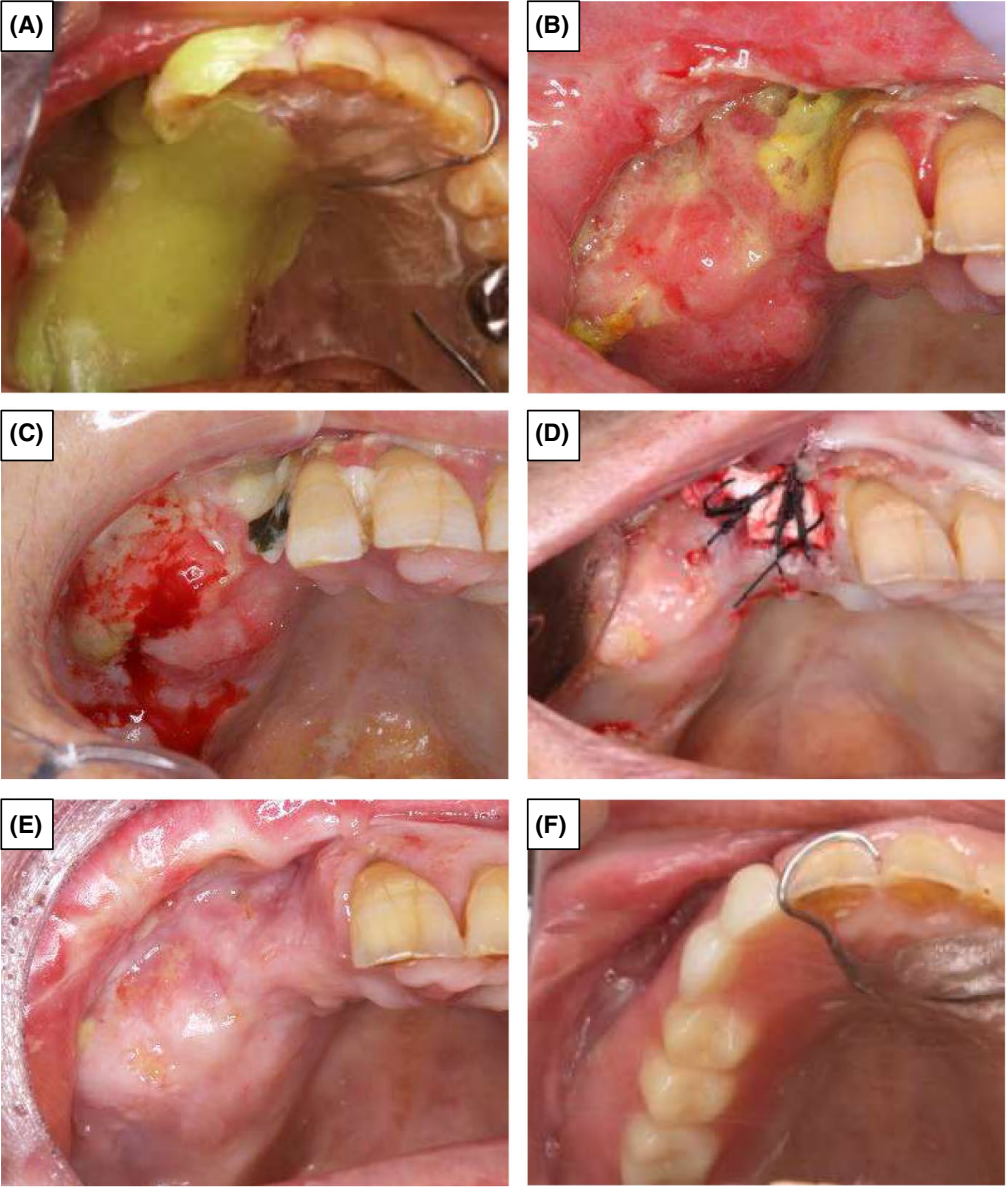
Treatment course after hospitalization. A, In addition to antimicrobial drip infusion, topical antibacterial drug (bacitracin, fradiomycin sulfate) delivery using a splint was started on hospital day 85. B, Exposed bone had undergone epithelization and protraction, and pain was under remission on hospital day 90. C, The right maxillary lesion with infection at the time of re‐hospitalization on day 115. D, Extraction of the mobile right lateral incisor and re‐saucerization with sharp‐edged maxillary bone were performed, and the open wound was covered with dermis defect grafting (Terudermis^®^) after acute symptomatic relief. E, The exposed bone underwent complete epithelization on hospital day 148. F, A denture combined with a protection plate was fabricated for oral intake of food

On hospital day 85, she was hospitalized because of poor pain control and difficulty in oral intake of food. We started 9 g/d of sulbactam/ampicillin (SBT/ABPC) with topical antibacterial drug (bacitracin, fradiomycin sulfate) delivery using a splint (Figure 4A) and professional oral management and gargling for acute infection from ARONJ. On hospital day 90, the acute symptoms had resolved. We extracted the mobile right canine and performed saucerization with exposure of the maxillary bone. The exposed bone had undergone epithelization and protraction, and pain was under remission. On hospital day 111, she was discharged (Figure 4B).

However, on day 115, she was re‐hospitalized because of fever, fatigue, infection of the right maxillary lesion, and difficulty in oral intake of food (Figure 4C). Her schizophrenia caused poor compliance to instructions, and therefore, she had not taken some prescribed drugs. We started her on SBT/ABPC again. However, the result of re‐culture of the lesion was persistent, so we switched to SBT/ABPC to 1.2 g/d of clindamycin phosphate. After acute symptoms were relieved, we extracted the mobile right lateral incisor and performed re‐saucerization with a sharp‐edged maxillary bone, and the open wound was covered with a dermis defect graft (Terudermis^®^) (Figure 4D). The exposure bone underwent complete epithelization on hospital day 148, and we prescribed a dosage of antibiotics (Figure 4E).

We fabricated the denture combined with a protection plate, which was good for oral intake of food (Figure 4F). On hospital day 160, she was discharged. There was no recurrence of MTX‐LPD or ARONJ, and RA remained well‐controlled. She undergoes regular follow‐ups every 3‐6 months.

## 3 DISCUSSION

Clinically, MTX‐LPD is similar to lymphoma in patients without immunodeficiency. However, both the immunological abnormalities of RA and the immunosuppressive effects of MTX are considered to be involved in the pathogenesis of MTX‐LPD. In RA, specific clonal autoreactive T cells are seen, and in their presence, B cells that produce autoantibodies such as rheumatoid factor are activated. Furthermore, it has been suggested that the immunosuppressive effects of MTX may cause viral infections or reactivation of latent viral infections and also induce abnormal cell proliferation.^7^, ^12^ Among viral infections, EBV is considered to be more common and is thought to cause lymphoma by direct viral activity that spreads and propagates the virus from latently infected cells.^13^, ^14^

Diffuse large B‐cell lymphoma (DLBCL) accounts for approximately half of all MTX‐LPD histological types, followed by Hodgkin lymphoma (HL) at 10%‐20%.^15^ Other studies have also mentioned follicular lymphoma and T‐cell lymphoma.^16^ Since these are not monoclonal lesions, it is necessary to make a comprehensive diagnosis by combining immunohistochemical staining and clinical features.

In this case as well, HE staining revealed Reed‐Sternberg‐like cells and Hodgkin cell‐like cells in the granulation tissue. Immunohistological staining revealed numerous CD20/30‐positive atypical large cells, which were EBER‐ISH positive. Since MTX was administered for a long duration of 20 years and the total dose was approximately 7680 mg, it was considered that immunosuppression and the proliferation of EBV‐infected cells occurred.

MTX‐LPD may resolve in several weeks after withdrawal of the MTX therapy.^17^ However, additional treatments, such as chemotherapy and radiotherapy, should be considered for patients with persistent LPD after MTX withdrawal.^18^ Therefore, definitive diagnostic criteria are lacking, and a definitive diagnosis of MTX‐LPD remains challenging. As in this case, if there are no lymph node lesions, and the diagnostic criteria for ONJ are met, there is a possibility of the misdiagnosis of ARONJ. Therefore, in cases of oral MTX administration, oral surgeons should bear in mind that incisional biopsy and immune‐histological examination are required for the final diagnosis.

Regarding the administration of antiresorptives to patients under ARONJ treatment, it is reported that their discontinuation may be recommended until ARONJ treatments are completed for patients with osteoporosis, excluding those with high fracture risk.^11^ Since this case was considered to be an advanced complication of MTX‐LPD and ARONJ, we consulted with the department of internal medicine and determined that the risk of fractures was not high. Therefore, we decided to withhold the antiresorptives after ARONJ treatment. In order to balance the risk of ARONJ and fractures, it was considered necessary to determine the discontinuation of antiresorptives after consultation with the medical department.

A review of the literature revealed 22 cases of intraoral localized MTX‐LPD with bone exposure, including our case (Table 1).^2^, ^3^, ^19^, ^20^, ^21^, ^22^, ^23^, ^24^, ^25^, ^26^, ^27^, ^28^, ^29^, ^30^, ^31^, ^32^, ^33^, ^34^, ^35^ The mean age of the patients was 73 years (range, 44‐92 years), and the male to female ratio was 2:1. The mean duration of MTX treatment was 8.6 years (range, 0.6‐20 years). The frequency of DLBCL was 84% (16/19 cases); the frequency of EBV infection was 95% (19/20 cases); MTX‐LPD resolved in 89% (16/18 cases) of patients after discontinuation of MTX. Data on the mean age, sex ratio, duration of MTX treatment, and frequency of EBV infection were similar to those of previous reports on intraoral localized MTX‐LPD.^35^ In contrast, the frequency of DLBCL was higher in comparison with previous reports on intraoral localized MTX‐LPD. Intraoral localized MTX‐LPD was considered to show good prognosis after drug withdrawal, with or without bone exposure. In three cases of no remission after withdrawal of MTX, chemotherapy was performed.^2^, ^23^, ^30^ Two of the three cases showed systemic symptoms,^2^, ^23^ and in one case, a fistula was detected in the palatal opening in the naso‐genian region.^30^ It was considered that the presence of extraoral symptoms had an influence on the prognosis of intraoral localized MTX‐LPD.

**Table 1:** Clinicopathological findings of 22 cases with intraoral localized methotrexate‐related lymphoproliferative disorders (MTX‐LPD) with bone exposure

	Case	Age, Sex	Site	Local findings	Systemic occurrence	Pathological diagnosis	EBV	Antiresorptive agent intake (y)	MTX intake (y)	Purulent discharge	Antibiotic therapy	MTX withdrawl	Chemo therapy	Recurrence
1	Kalantzis et al^12^	72, F	U. gingiva	Ulceration Bone exposure	UK	Polyclonal B‐cell lesion	+	−	UK	−	−	+	−	−
2	Acero et al^2^	79, F	U. gingiva	Pain Ulceration Bone exposure	−	DLBCL	+	−	15 y	+	−	+	+	−
3	Tanaka et al^3^ ^,^ ^a^	44, M	U. gingiva	Pain Ulceration Bone exposure Unhealed tooth socket	−	DLBCL	+	−	UK	−	−	+	−	−
4	Hatanaka et al^13^ ^,^ ^a^	66, F	U. gingiva	Pain Ulceration Bone exposure	−	DLBCL	+	−	12 y	+	+	+	−	−
5	Yamashita et al^14^ ^,^ ^a^	66, F	U. gingiva	Pain Bone exposure Unhealed tooth socket	−	DLBCL	+	4 y	12 y	−	+	+	−	−
6	Sano et al^15^ ^,^ ^a^	70, M	U and L. gingiva	Pain Ulceration Bone exposure	−	DLBCL	+	−	0.4 y	−	+	+	−	−
7	Minami et al^16^ ^,^ ^a^	60, F	L. gingiva	Pain Swelling Bone exposure	−	UK	+	3 y	2 y	−	+	+	+	+
8	Kimoto et al^17^ ^,^ ^a^	64, F	U and L. gingiva	Pain Ulceration Bone exposure	−	DLBCL	+	2 y	3 y	−	+	+	−	−
9	Aiko and Michiwaki 2013^18^ ^,^ ^a^	84, F	L. gingiva	Pain Swelling Ulceration Bone exposure	−	DLBCL	+	−	1.6 y	−	+	+	−	−
10	Goto et al^19^ ^,^ ^a^	74, F	U. gingiva	Ulceration Bone exposure Unhealed tooth socket	Lung Pharyngeal Tonsil	Polymorphic LPD	+	−	10 y	−	−	+	−	−
11	Horie et al^20^ ^,^ ^a^	60, M	U. gingiva	Ulceration Bone exposure Unhealed tooth socket	−	DLBCL	+	−	12 y	−	+	+	−	−
12	Yagihara et al^21^ ^,^ ^a^	87, M	U. gingiva	Ulceration Bone exposure	−	T‐cell dominant polymorphic LPD	−	5 y	12 y	−	+	+	−	−
13	Obata et al^22^ ^,^ ^a^	80, M	U. gingiva	Pain Ulceration Bone exposure	−	DLBCL	+	−	6.3 y	−	−	+	−	−
14	Saito et al^23^ ^,^ ^a^	76, F	U. gingiva	Bleeding Ulceration Bone exposure	Lung Stomach	DLBCL	+	−	10 y	−	−	+	+	+
15	Mese et al^24^ ^,^ ^a^	70, F	U. gingiva	Pain Swelling Ulceration Bone exposure	−	DLBCL	+	−	15 y	−	+	+	−	−
16	Miyamoto et al^25^ ^,^ ^a^	72, F	U. gingiva	Pain Ulceration Bone exposure	−	DLBCL	+	0.5 y	8 y	−	+	+	−	−
17	Komatani et al^26^ ^,^ ^a^	88, M	U. gingiva	Bone exposure Unhealed tooth socket	−	UK	UK	−	0.6 y	+	+	−	−	No remission
18	Komatani et al^26^ ^,^ ^a^	73, M	L. gingiva	Pain Ulceration Bone exposure	−	UK	UK	−	2 y	−	+	+	−	Not available
19	Hatakeyama et al^27^ ^,^ ^a^	92, F	L. gingiva	Pain Ulceration Bone exposure	−	DLBCL	+	−	13 y	−	−	+	−	Not available
20	Hatakeyama et al^27^ ^,^ ^a^	80, F	U. gingiva	Pain Ulceration Bone exposure	Lung Pharyngeal	DLBCL	+	−	13 y	−	−	+	−	Not available
21	Furukawa et al^28^ ^,^ ^a^	81, F	U. gingiva	Pain Bleeding Ulceration Bone exposure	−	DLBCL	+	UK	4 y	−	−	+	−	−
22	Our case	75, F	U.gigiva	Pain Bleeding Ulceration Bone exposure	−	DLBCL	+	5 y	20 y	+	+	+	−	−

Abbreviations: C, chemotherapy; DLBCL, diffuse large B‐cell lymphoma; EBV, Epstein‐Barr virus; L, lower jaw; LPD, lymphoproliferative disorder; U, upper jaw; UK, unknown; W, withdrawal of MTX; y, yrars.

^a^ Japanese patient.

Most intraoral localized MTX‐LPD patients (20/22) were Japanese.^3^, ^20^, ^21^, ^22^, ^23^, ^24^, ^25^, ^26^, ^27^, ^28^, ^29^, ^30^, ^33^, ^35^ However, the reason is unclear. Therefore, a study has been initiated jointly by three academic societies (Japan College of Rheumatology, The Japanese Society of Hematology, The Japanese Society of Pathology) in Japan.

In this case, it was necessary to administer antimicrobials during hospitalization because of evacuation in the exposed bone, fever, fatigue, elevation of the inflammatory markers, and an eating disorder caused by pain. Although symptom relief was observed after MTX withdrawal, bone exposure was continued, and reinfection of the bone exposed area was observed because of interruption of the antibiotic. Therefore, taking into consideration the combination of ARONJ, orthopedic treatment with dermis defect grafting and topical antibacterial drug delivery using a splint were actively performed in addition to antibiotic instillation, and the bone exposure improved, and the lesion healed.

Twenty‐two cases of intraoral localized MTX‐LPD with bone exposure have been reported, but only four cases had infection with purulent discharge.^2^, ^33^, ^34^ As bone exposure because of LPD is caused by gingival necrosis due to lymphocyte infiltration, and infection is considered to be another pathology, there might be only four cases of true ARONJ complication where infection could not be controlled unless antibacterial drugs were used. Our case had severe infections repeatedly in a short time period; therefore, it was considered that LPD was complicated by ARONJ.

## 4 CONCLUSION

In cases with a history of oral MTX and presence of oral ulcers, it is important to perform biopsy and immunostaining for LPD‐related markers, even if they meet the diagnostic criteria for ARONJ. In addition, when bone exposure involves bacterial infection, aggressive antibiotic administration and surgical treatment are considered important to promote epithelialization of the wound.

## CONFLICT OF INTEREST

The authors have no conflicts of interest to disclose.

## AUTHOR CONTRIBUTIONS

MM, TS, and KM: managed the patient. KH: made the pathological diagnosis. MM, TS, and TN: wrote the manuscript. All authors: reviewed and approved the final version of the manuscript.

## ETHICAL APPROVAL

Ethical Approval was not necessary for this case report. Patient's data and photographs are de‐identified.
